# Dual-Use Quickscan: A Web-Based Tool to Assess the Dual-Use Potential of Life Science Research

**DOI:** 10.3389/fbioe.2021.797076

**Published:** 2021-12-09

**Authors:** Iris M. Vennis, Mirjam M. Schaap, Petra A. M. Hogervorst, Arnout de Bruin, Sjors Schulpen, Marijke A. Boot, Mark W. J. van Passel, Saskia A. Rutjes, Diederik A. Bleijs

**Affiliations:** Biosecurity Office, National Institute for Public Health and the Environment, Bilthoven, Netherlands

**Keywords:** dual-use research of concern, misuse, biosafety, biosecurity, biorisk management, assessment tool, high-risk pathogens, international health regulations

## Abstract

Research on pathogenic organisms is crucial for medical, biological and agricultural developments. However, biological agents as well as associated knowledge and techniques, can also be misused, for example for the development of biological weapons. Potential malicious use of well-intended research, referred to as “dual-use research”, poses a threat to public health and the environment. There are various international resources providing frameworks to assess dual-use potential of the research concerned. However, concrete instructions for researchers on how to perform a dual-use risk assessment is largely lacking. The international need for practical dual-use monitoring and risk assessment instructions, in addition to the need to raise awareness among scientists about potential dual-use aspects of their research has been identified over the last years by the Netherlands Biosecurity Office, through consulting national and international biorisk stakeholders. We identified that Biorisk Management Advisors and researchers need a practical tool to facilitate a dual-use assessment on their specific research. Therefore, the Netherlands Biosecurity Office developed a web-based Dual-Use Quickscan (www.dualusequickscan.com), that can be used periodically by researchers working with microorganisms to assess potential dual-use risks of their research by answering a set of fifteen yes/no questions. The questions for the tool were extracted from existing international open resources, and categorized into three themes: characteristics of the biological agent, knowledge and technology about the biological agent, and consequences of misuse. The results of the Quickscan provide the researcher with an indication of the dual-use potential of the research and can be used as a basis for further discussions with a Biorisk Management Advisor. The Dual-Use Quickscan can be embedded in a broader system of biosafety and biosecurity that includes dual-use monitoring and awareness within organizations. Increased international attention to examine pathogens with pandemic potential has been enhanced by the current COVID-19 pandemic, hence monitoring of dual-use potential urgently needs to be encouraged.

## Introduction

Research on pathogenic organisms is crucial for innovations in the medical, biological and agricultural fields. To ensure safe and secure research, there are biorisk management guidelines for research organizations, including hospitals, biotechnology companies, and universities (ISO, 2019). These guidelines include both biosafety measures, focusing on preventing unintentional release of hazardous biological agents (WHO, 2020a), and biosecurity measures, focusing on preventing intentional release of biological agents (WHO, 2006). Though, research on biological agents, including information and techniques developed to improve health, welfare, and safety, can also be misused for harmful purposes with potential public health, ecological, economical, and societal consequences. Well-intended research with potential for malicious use is been referred to as dual-use research and poses a threat to public health and the environment. Examples thereof include yeast strains converting sugars into opiates, paving the way for homemade heroin (Ehrenberg, 2015) or reconstruction of a pandemic virus, that could be misused by malicious actors as a biological weapon (Tumpey et al., 2005). However, dual-use awareness amongst life science researchers remains a topic that needs continuous attention (NASEM, 2017; Sarwar et al., 2019). In today’s society, anyone can obtain online information about science and technology relatively easily. Researchers are responsible for the information they provide and have a duty to prevent misuse of their research (KNAW, 2009). Therefore, increased awareness of biosecurity and dual-use among researchers is crucial and dual-use potential should be assessed (KNAW, 2009; iGEM Team Bielefeld, 2015; EBRF, 2016; NASEM, 2018; IWG, 2020; IWG, 2021). This was also one of the conclusions of the 2004 report *Biotechnology Research in an Age of Terrorism* of the National Research Council (NRC, 2004). The report recommended to create an expert committee to provide advice, guidance, and leadership for a system of review and oversight of experiments of concern, which led to the establishment of the National Science Advisory Board for Biosecurity in the United States in 2005. Biosecurity and dual-use receive increasing international attention. Subsequently, WHO recently published a report on the findings of an international horizon scan on dual-use research of concern in the life sciences (WHO, 2021) and the International Health Regulations (IHR) benchmarks describe countries need to *develop documents for dual-use research* in order to achieve a demonstrated capacity in biosafety and biosecurity (WHO, 2019). Several countries developed national guidelines on dual-use and responsible science (German Ethics Council, 2014; DURC Policy, 2014a; PHAC, 2018), including the Royal Netherlands Academy of Arts and Sciences’ (KNAW) report *Improving biosecurity, Assessment of dual-use research* (KNAW, 2013) and *Guidelines for researchers on dual-use and preventing misuse of research* published by a collaboration of five Flemish universities (Flemish Interuniversity Council, 2017). Nevertheless, in the past decade several publications have raised concerns with health security experts and government authorities. This includes concerns about publishing knowledge about the gain of mammalian transmissibility for influenza A/H5N1 virus (Herfst et al., 2012; Imai et al., 2012) and publication of the methods for the synthesis of a viable and infectious horsepox virus, a virus related to smallpox (Noyce et al., 2018). The dual-use potential of these papers was often discussed at the very end of a research cycle, as authorities assess flagged research with dual-use concerns often only during the publication process. Furthermore, the last decade saw a sharp increase in the number of high-containment biological laboratories in order to do more research on and improve our understanding of new and re-emerging dangerous pathogens (Lentzos and Koblentz, 2021). However the possibility of accidents, thefts or malicious use increases with each additional laboratory and the degree of oversight and control varies (Peters, 2018). The current COVID-19 pandemic leads to even more international attention to examine pathogens with pandemic potential (Grange et al., 2021) and causes Gain-of-Function experiments to be reconsidered (Imperiale and Casadevall, 2020). This highlights the urgent need for better assessment of potential dual-use research of concern (Jonas et al., 2020). The 2004 report of the National Research Council mentions seven types of experiments and fifteen pathogens that can be labeled as dual-use research of concern (NRC, 2004). However, there are more types of experiments and pathogens that could possibly also lead to dual-use concerns (Wintle et al., 2017). Concrete instructions and clear guidance for researchers on how to perform a dual-use risk assessment is lacking. A need for practical dual-use monitoring and risk assessment instructions, in addition to the need to raise awareness among scientists about potential dual-use aspects of their research was internationally recognized by amongst other the Global Health Security Agenda action package Biosecurity and Biosafety (GHSA, 2020), the international working group on strengthening the culture of biosafety and biosecurity (IWG, 2021), and the Global Biosecurity Dialogue (NTI, 2021).

To meet these needs, the Netherlands Biosecurity Office developed a web-based tool to identify potential dual-use aspects in research. This Dual-Use Quickscan consists of 15 questions about different aspects of research that may affect dual-use potential. Researchers working with microorganisms can use the Quickscan prior to the start of their research as well as periodically to assess potential dual-use risks of their research. The results of the Quickscan provide the researcher with an indication of possible dual-use potential of their research and can be used as a basis for further discussion with a Biorisk Management Advisor. Biorisk Management Advisors are staff, such as biological safety officers, consultant microbiologists, occupational hygienists, or safety personnel, designated to provide advice, guidance, and assurance on biorisk management issues as described in ISO 35001:2019 (ISO, 2019). Assessment throughout the research cycle, at the start, during and at the end of a research project, enables timely management of the dual-use character to ensure that research will progress in a safe and secure way and publication is not hampered. In addition, this tool contributes to stimulate dual-use awareness among researchers. This paper describes the development, application, and implementation of the Dual-Use Quickscan.

## Development

For the development of the Dual-Use Quickscan an extensive literature search was performed to identify existing documents dealing with frameworks to assess dual-use potential of the research concerned. Areas to assess were identified from a broad range of literature (Table 1), including the report *Biotechnology Research in an Age of Terrorism* of the National Research Council (NRC, 2004), the report *Improving biosecurity, Assessment of dual-use research* by the Royal Netherlands Academy of Arts and Sciences (KNAW) (KNAW, 2013), and *United States Government Policy for Institutional Oversight of Life Sciences Dual Use Research of Concern* (DURC Policy, 2014a). Questions from existing dual-use assessment frameworks were extracted and grouped by theme. A team of biorisk experts assessed the relevance of the themes and formulated yes/no questions to assess dual-use characteristics of research corresponding to the themes. The questions are formulated in such a way to stimulate discussion and increase awareness of possible dual-use aspects of scientific research. To meet the need of a clear, concise, to-the-point assessment tool, only the most important dual-use aspects were selected for the Quickscan. For better understanding of the question, the team of biosecurity experts provided each question with an explanation and some typical examples from literature that demonstrate the corresponding dual-use characteristics in research. Each literature example was given a title reflecting the dual-use characteristic, an explanation of the dual-use aspect, and a summary of the study. A citation and link are provided for further reading. The formulated questions can be grouped into three categories: 1) Characteristics of biological agent such as virulence, production rate, transmission, distribution, tropism, availability of medical countermeasures, and resistance to clinically relevant medical countermeasures or to other characteristics that may make the agent interesting as a biological warfare agent, 2) Knowledge and technology about the biological agent, relating to knowledge, methods and technologies, and 3) Consequences of misuse, concerned with the possible consequences of misuse in the field of ecology, economy and society.

**TABLE 1:** Overview of literature used to extract questions and areas to assess for the Dual-Use Quickscan.

Author/Organization	Year	Titel	References
Boston University	2014	Identifying and Addressing Dual Use Research of Concern	DURC Boston University (2014)
Canadian Government	2018	Canadian Biosafety Guideline—Dual-Use in Life Science Research	PHAC (2018)
Centre for Biosecurity and Biopreparedness, Denmark	2015	Questionnaire about dual-use research of concern for companies, project managers etc.	CBB (2015)
German Ethics Council	2014	Biosecurity Freedom and Responsibility of Research	German Ethics Council (2014)
Federation of American Scientist		Case studies Dual-use	FAS (2016)
iGEM Team Bielefeld-CeBiTec	2015	Dual Use report	iGEM Team Bielefeld (2015)
Imperiale MJ, Casadevall A	2015	A new synthesis for dual use research of concern	Imperiale and Casadevall (2015)
ISO	2019	ISO 35001:2019, Biorisk management for laboratories and other related organisations	ISO (2019)
Jonathan B. Tucker	2012	Innovation, Dual Use, and Security. Managing the Risks of Emerging Biological and Chemical Technologies	Tucker (2012)
National Academies of Sciences, US	2018	Governance of Dual-use Research in the Life Sciences: Advancing Global Consensus on Research Oversight: Proceedings of a Workshop	NASEM (2018)
National Institutes of Health, US	2014	Tools for the Identification, Assessment, Management, and Responsible Communication of Dual Use Research of Concern. A Companion Guide to the United States Government Policies for Oversight of Life Sciences Dual Use Research of Concern	DURC Tools (2014)
National Institutes of Health, US	2014	Implementation of the USG Policy for Institutional Oversight of Life Sciences DURC: Illustrative case Studies	DURC Policy (2014b)
National Institutes of Health, US		Dual Use Research of Concern	NIH (2021)
National Research Council, US	2004	Biotechnology Research in an Age of Terrorism	NRC (2004)
National Research Council, US	2007	Science and Security in a Post 9/11 World: A Report Based on Regional Discussions Between the Science and Security Communities	NRC (2007)
Robert Koch Institute (RKI), Germany	2013	Handling Dual-use Risks at the RKI - House Order_ Dual-Use Potential in Research	RKI (2013)
Royal Netherlands Academy of Arts and Sciences (KNAW)	2013	Improving biosecurity: Assessment of dual-use research	KNAW (2013)
Selgelid MJ.	2009	Governance of dual-use research: an ethical dilemma	Selgelid (2009)
United States Government	2014	United States Government Policy for Institutional Oversight of Life Sciences Dual use Research of Concern	DURC Policy (2014a)
Whitby S, Novossiolova T, Walther G and Dando M	2015	Preventing Biological Threats: What You Can Do. A Guide to Biological Security Issues and How to Address Them	Whitby et al. (2015)
Working Group Dual-use of the Flemish Interuniversity Council	2017	Guidelines for researchers on dual-use and misuse of research	Flemish Interuniversity Council (2017)
World Health Organization (WHO)	2020	Laboratory Biosafety Manual 4th Edition; Biosafety programme management	WHO (2020b)

To check for practical applicability, correctness and ease of use, the Dual-Use Quickscan was reviewed by an expert committee consisting of renowned researchers, biological safety officers and safety experts from academia, industry and government. The expert committee was requested to provide feedback on the content, design, scope and relevance of the Dual-Use Quickscan. This included assessing if the formulated questions include all important aspects for assessing dual-use research, if themes are missing or superfluous, and if there are sources missing that should be consulted for the development of the Quickscan. The expert committee also provided feedback on correct and understandable wording of the questions and explanations. The feedback of the reviewers was incorporated, leading to the final Dual-Use Quickscan consisting of 15 questions.

Finally, the Dual-Use Quickscan was made freely available as a web-based tool at www.dualusequickscan.com. For security reasons, the web-based tool is filled out anonymously and no data of entered fields or results are saved. The data entered in this tool will not be sent via the Internet and is not stored by the Biosecurity Office. Data are stored locally on the user’s computer only using cookies. This way, it is possible to complete the Dual-Use Quickscan at another time. The entered data can be deleted, for instance to repeat the Dual-Use Quickscan, by a build-in “Clear data” button. The results can be stored for the researcher’s own administration by saving the results locally as PDF.

## Application

The Dual-Use Quickscan is a web-based tool and consists of 15 contextualized questions about different aspects of research that may contribute to dual-use potential. The themes and questions included in the Dual-Use Quickscan are displayed in Table 2. The questions concern not only the biological agent, including bacteria and viruses, but also toxins produced or derived from it. Furthermore, the Quickscan is not limited to human pathogens, but also focuses on animal and plant pathogens. For example gene drives could be used for malicious purposes, such as altering populations of agricultural plants or livestock with harmful intents (Oye et al., 2014). As dual-use risks are not only associated with high-risk pathogens, the questions also relate to lower classified pathogens or research with harmful consequences for ecology, economy or society. Examples thereof include yeast strains converting sugars into opiates, paving the way for homemade heroin (Ehrenberg, 2015) and bacteria that can break down metals, whereby such biological agents could also be used to destroy working electronics (IGEM, 2018). Literature examples are provided for each question. An overview of all literature examples included in the Dual-Use Quickscan is also presented in Table 2. Each question can be answered with: yes, no or unknown. For a complete dual-use assessment overview, all questions will need to be answered.

**TABLE 2:** The 15 themes and corresponding questions of the Dual-Use Quickscan.

Question nr.	Theme	Question	Literature examples
1	High-risk biological agent	Are you working with a biological agent, or parts of it, that can be considered a high-risk pathogen?	Pohanka and Kuča (2010), Cieslak et al. (2018)
2	Host range and tropism	Is the host range or tropism of the biological agent likely to be altered?	Dmitriev et al. (1998), Menachery et al. (2015)
3	Virulence	May your research increase the virulence of the biological agent?	Mellata et al. (2010), Dowall et al. (2014)
4	Stability	Is it to be expected that the stability of the biological agent outside the host will increase as a result of your research?	Nguyen et al. (2019), Doekhie et al. (2020)
5	Transmissibility	Is it likely that the transmissibility or ability for dispersion or dissemination of the biological agent will increase?	Herfst et al. (2012), Imai et al. (2012)
6	Absorption and toxicokinetics	Is it to be expected that the absorption of the biological agent is facilitated or is an increased toxicokinetic effect to be expected?	Wollert et al. (2007), Wang et al. (2020)
7	Drug resistance	Is it likely that your research will increase the resistance of the biological agent to clinical and/or agricultural prophylactic or therapeutic interventions, including antimicrobial resistance?	Pomerantsev et al. (1997), Kerr et al. (2004), Udani and Levy (2006)
8	Population immunity	Does the biological agent possibly have a negative effect on the immunity of humans, animals or plants?	Rosengard et al. (2002), Sweere et al. (2019)
9	Detection methodology and diagnostics	Could your research impact the detection methods, diagnostics, or clinical diagnosis of the biological agent?	Anisimov (1999), Zilinskas (2017)
10	Reconstruction	Does your research contribute to the reconstruction of an eradicated or extinct biological agent?	Cello et al. (2002), Tumpey et al. (2005), Dewannieux et al. (2006)
11	Harmful effects	May changes to the biological agent possibly generate or enhance the harmful consequences, which may involve “improved weaponization"?	Edwards et al. (1997), Dover et al. (2014), Dowall et al. (2014)
12	Knowledge and Technology	Is it likely that the knowledge you obtain and technologies you develop in your research allow others to use them for malicious purposes?	Noyce et al. (2018), Baselga-Cervera et al. (2019), Lewis et al. (2019), Thi Nhu Thao et al. (2020)
13	Ecological consequences	Could your research contribute to possible harmful ecological consequences due to misuse of the modified biological agent or the knowledge thereof?	Oye et al. (2014), Reeves et al. (2018), Scudellari (2019)
14	Economic consequences	Could your research contribute to possible harmful economic consequences due to misuse of the modified biological agent or the knowledge thereof?	Suffert et al. (2009), Reeves et al. (2018)
15	Consequences for society	Could your research contribute to harmful consequences for society from the misuse of the modified biological agent or the knowledge thereof?	Ehrenberg (2015); Fossati et al. (2015), Baselga-Cervera et al. (2019)

The results of the Quickscan provide a general representation of the dual-use potential of the research concerned. The outcome will lead to one of three interpretations. 1) One or more questions are filled in with “yes”. The more questions are filled in with “yes”, the more likely it is that the research contains dual-use characteristics. 2) One or more questions are answered with “unknown”, indicating that at the time of completing the questionnaire, it is not clear whether associated dual-use aspects may be present, but this may change during the course of the research studies. 3) If all questions are answered with “no”, it is unlikely that aspects of dual-use potential are associated with the study, but this cannot be ruled out. When one or more questions have been answered by “yes” or “unknown”, it is important to discuss the outcome with a Biorisk Management Advisor. The questions are intended to raise awareness on potential dual-use aspects of the research and therefore can form the starting point for a discussion on dual-use potential of the research and how to deal with this. In addition, the results can also be discussed with direct colleagues aiming to create awareness about potential dual-use aspects of the research. The results of the Quickscan are made available to the user as a PDF document. It should be noted that the answers to the Quickscan reflect the current situation only. During the course of the study, it may occur that questions will be answered differently, depending on e.g., the results achieved by the study. Therefore, it is important to use the Quickscan periodically to revise whether the right biosecurity measures are in place, e.g., using a plan-do-check-act cycle as described in ISO 35001:2019 (ISO, 2019).

## Implementation

**FIGURE 1:**
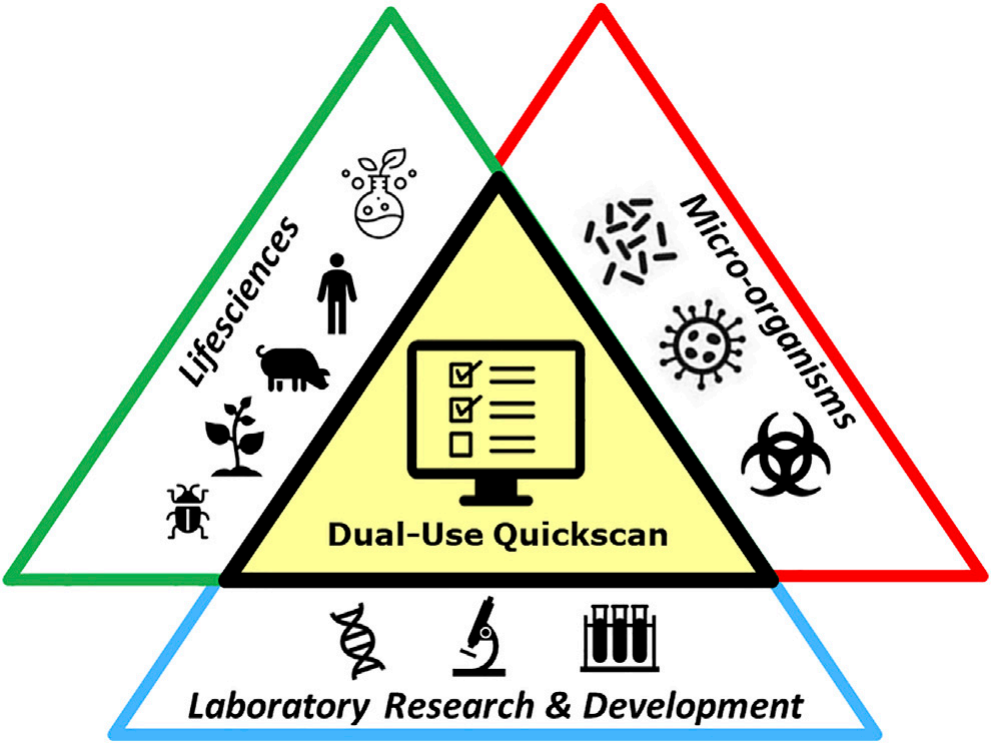
Graphical display of the target users of the Dual-Use Quickscan. The Quickscan is developed for users employed in the field of life sciences, working with (parts or products of, or knowledge on) microorganisms, and performing (laboratory) activities for research, development or production processes.

The Dual-Use Quickscan has been developed for people employed in the field of life sciences, who are working with (parts or products of, or knowledge on) microorganisms, and perform (laboratory) activities for research, development or production processes (Figure 1). The life-sciences include, but is not limited to the field of physiology, neurobiology, cell biology, developmental biology, ecology, evolutionary biology, microbiology, virology, plant biology, bioinformatics, synthetic biology, or nano- or molecular biology. A Biorisk Management Advisor, as described in ISO 35001:2019 (ISO, 2019), could advise to whom the Dual-Use Quickscan is applicable. The scope of the Dual-Use Quickscan covers the entire research, development or production process and is not limited to a single separate experiment, but might be applicable for the research program of the entire department. The organization is responsible for Biorisk management and should calculate the interval of the Dual-Use Quickscan. This might differ per institute and could depend on various factors, such as the nature of the research or other dual-use considerations. In general, completing the Dual-Use Quickscan is useful for new research (for example when applying for a grant), in case of important changes to a current research project, or when any unforeseen results of the research occur, or prior to publication.

**FIGURE 2:**
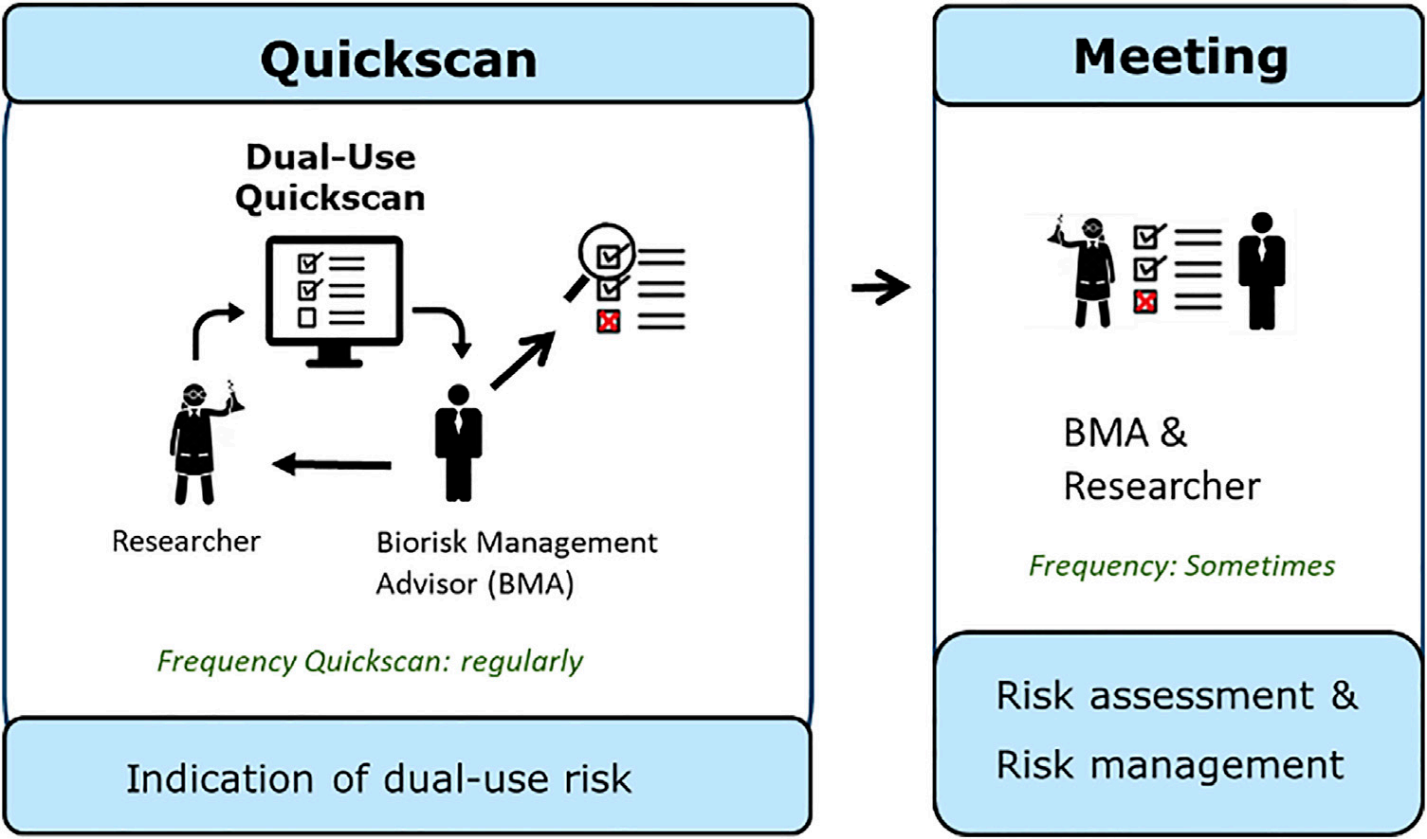
Graphical representation of the steps using the Dual-Use Quickscan and meeting with a Biorisk Management Advisor (BMA) for further assessment. Researchers are requested by a BMA or decide themselves to fill in the Dual-Use Quickscan. The researcher provides the results of the Quickscan to the BMA. This cycle is performed regularly. In case the Quickscan gives an indication of dual-use potential, the BMA and researcher should have a meeting to further discuss the results of the Quickscan, to perform a risk assessment and manage the risks. This meeting only has to take place in some cases.

After completion of the Quickscan, the first screening on dual-use potential is completed. The researcher shares the results of the Quickscan with the Biorisk Management Advisor, who in some cases may decide to discuss the outcome with the researcher to gain more in-depth information about the results of the Dual-Use Quickscan and to further assess the potential dual-use characteristics of research concerned. In this next phase (Figure 2), a risk assessment can be performed possibly leading to measures to ensure that the research progress is not hampered and to manage the dual-use characteristics of the research concerned. In this case the Biorisk Management Advisor and researcher together develop a BioRisk Mitigation Plan. After implementation of mitigation measures, the research will be reviewed. If the measures are sufficient, the plan can be approved and critical projects are allowed to continue to enhance scientific knowledge. If no unambiguous solution or conclusion can be found, or if there is still doubt on the possible dual-use nature, a consultation at institute level can be used, for example with the Biorisk Management Committee, as described in ISO 35001: 2019 (ISO, 2019). This committee may consist of researchers, the person responsible for biological safety, top management, and possibly supplemented with other disciplines (e.g., virologists or ethicists). The discussion can continue in this committee to evaluate the research project in order to manage the potential dual-use character of the research. In these meetings, risks can be assessed based on models from literature, such as Tucker’s model (Tucker, 2012); a decision making framework for ethical questions. Considerations described within this model can be helpful in this regard, and are also mentioned in the KNAW report Improving biosecurity, Assessment of dual-use research (KNAW, 2013). The companion guide titled Tools for the Identification, Assessment, Management, and Responsible Communication of Dual Use Research of Concern and the United States Government Policy for Institutional Oversight of Life Sciences Dual Use Research of Concern also offers guidelines for further assessment of the dual-use potential of research (DURC Tools, 2014; DURC Policy, 2014a).

## Discussion

The Dual-Use Quickscan addresses the international need for a clear and practical dual-use assessment for life science researchers. The current COVID-19 pandemic accelerates the need for better, practical dual-use assessment. Using a synthetic genomics platform, the SARS-CoV-2 virus was rapidly reconstructed and shared (Thi Nhu Thao et al., 2020). This synthetic genomics platform can be used to create viruses with large complex genomes within as little as a week. The dual-use nature of this technology might also pose biosecurity and dual-use risks to our society (Gao et al., 2020), such as the deliberate spread of dangerous viruses by malicious actors. In addition, the SARS-CoV-2 pandemic has led to an increased interest in the study of coronaviruses and other biological agents with pandemic potential. It is of vital importance for public health and society to generate and share new and important knowledge on the emerging pathogens. However, the risks of potential misuse should also be well assessed. The Joint External Evaluation (JEE) developed by the World Health Organization (WHO) describes for the indicator biosafety and biosecurity a target including securing and monitoring dangerous pathogens and reducing dual-use risks (IHR, 2005). An important aspect to assess this is whether there is a mechanism for biosecurity oversight of dual-use research. The WHO Laboratory Biosafety Manual monograph *Biosafety programme management* places emphasis on biosecurity oversight and assessment, including dual-use potential (WHO, 2020b). The Dual-Use Quickscan aims to close the gap in dual-use assessment and can be the first step towards structural assessment to increase oversight. The Quickscan should be placed in a broader risk management system including biosafety, biosecurity, dual-use monitoring and awareness within organizations. Although the Dual-Use Quickscan focuses on potential dual-use characteristics of the research itself, researchers should also be aware of collaborating partners, their role and their interests, and the funder(s) of research, not only nationally but also internationally. To generate a safe and secure culture of biosecurity, organizations need to work on the eight pillars of good biosecurity practice: Biosecurity awareness, Personnel reliability, Transport security, Information security, Accountability for materials, Emergency response, Management, and Physical security and assess these pillars (Sijnesael et al., 2014; Meulenbelt et al., 2019). There are tools available to assess laboratory biosafety and biosecurity risks, such as the Biosecurity Checklist developed by the Association of Public Health Laboratories (APHL) (APHL, 2019) and the Biosecurity Checklist for Laboratory Assessment and Monitoring (Brizee et al., 2019). Furthermore, the Biosecurity Resource Toolbox containing both biosecurity and dual-use resources and tools, available on the website of the European Biosecurity Regulators Forum (EBRF), may provide guidance on good biosecurity practice (EBRF, 2020). Many of these tools are complimentary in a full institutional biorisk approach.

## Conclusion

This paper describes a web-based tool to assess the dual-use potential of life science research. The aim of the tool is to provide a clear and practical dual-use risk assessment of research concerned and to create dual-use awareness amongst life-science researchers, as explicitly stated in the WHO Benchmarks for International Health Regulations Capacities. The results provide an indication of the dual-use potential and offers advice on the need of further assessment in consultation with a Biorisk Management advisor. The Dual-Use Quickscan can be embedded in a broader Biorisk management system within organizations.

## Authors Disclaimer

This product has been developed by the Netherlands Biosecurity Office on the basis of current scientific insights in the field of dual-use research. A negative score for all questions is no guarantee that there are no dual-use aspects of the research. The Dual-Use Quickscan is intended to encourage researchers to think about dual-use potential of the research concerned and to be used as a starting point for a discussion on the topic within an organization. The developers of this product assume no responsibility for errors or inaccuracies in the Dual-Use Quickscan, or its use. The Biosecurity Office also accepts no liability for damage caused by the use of the Dual-Use Quickscan, downloading the final report, and the data in the final report. The Quickscan is anonymous and data is not uploaded or shared with the Biosecurity Office. The web tool is freely available at www.dualusequickscan.com. If you have any questions or for more information, please contact the Biosecurity Office: biosecurity@rivm.nl.

## References

[B3] Anisimov A. (1999). Molecular-genetic Mechanisms of the Formation and Functional Significance of the Capsule of Yersinia pestis. Unpublished dissertation. 10.13140/2.1.4919.8088

[B1] APHL (2019). A Biosecurity Checklist: Developing A Culture of Biosafety and Biosecurity: The Association of Public Health Laboratories (APHL). Available from: https://www.aphl.org/courses/Documents/PHLTC%202019/Clinical_Lab_Biosecurity_Checklist%20Final.pdf .

[B4] Baselga-Cervera B. García-Balboa C. López-Rodas V. Fernández Díaz M. Costas E. (2019). Evidence of Microalgal Isotopic Fractionation through Enrichment of Depleted Uranium. Sci. Rep. 9 (1), 1973. 10.1038/s41598-019-38740-2 30760845 PMC6374374

[B8] Brizee S. Passel M. W. J. V. Berg L. M. V. D. Feakes D. Izar A. Lin K. T. B. (2019). Development of a Biosecurity Checklist for Laboratory Assessment and Monitoring. Appl. Biosaf. 24 (2), 83–89. 10.1177/1535676019838077 32655326 PMC7323818

[B5] CBB (2015). Questionnaire about Dual-Use Research of Concern for Companies, Project Managers Etc. Denmark: Centre for Biosecurity and Biopreparedness. Available from: https://www.biosecurity.dk/fileadmin/user_upload/PDF_FILER/UK_forms_and_guides/Questionnaire_about_dual-use_research_of_concern.pdf .

[B11] Cello J. Paul A. V. Wimmer E. (2002). Chemical Synthesis of Poliovirus cDNA: Generation of Infectious Virus in the Absence of Natural Template. Science 297 (5583), 1016–1018. 10.1126/science.1072266 12114528

[B12] Cieslak T. J. Kortepeter M. G. Wojtyk R. J. Jansen H. J. Reyes R. A. Smith J. O. (2018). Beyond the Dirty Dozen: A Proposed Methodology for Assessing Future Bioweapon Threats. Mil. Med. 183 (1-2), e59–e65. 10.1093/milmed/usx004 29401327

[B14] Dewannieux M. Harper F. Richaud A. Letzelter C. Ribet D. Pierron G. (2006). Identification of an Infectious Progenitor for the Multiple-Copy HERV-K Human Endogenous Retroelements. Genome Res. 16 (12), 1548–1556. 10.1101/gr.5565706 17077319 PMC1665638

[B15] Dmitriev I. Krasnykh V. Miller C. R. Wang M. Kashentseva E. Mikheeva G. (1998). An Adenovirus Vector with Genetically Modified Fibers Demonstrates Expanded Tropism via Utilization of a Coxsackievirus and Adenovirus Receptor-independent Cell Entry Mechanism. J. Virol. 72 (12), 9706–9713. 10.1128/jvi.72.12.9706-9713.1998 9811704 PMC110480

[B16] Doekhie A. Dattani R. Chen Y.-C. Yang Y. Smith A. Silve A. P. (2020). Ensilicated Tetanus Antigen Retains Immunogenicity: *In Vivo* Study and Time-Resolved SAXS Characterization. Sci. Rep. 10 (1), 9243. 10.1038/s41598-020-65876-3 32513957 PMC7280242

[B17] Dover N. Barash J. R. Hill K. K. Xie G. Arnon S. S. (2014). Molecular Characterization of a Novel Botulinum Neurotoxin Type H Gene. J. Infect. Dis. 209 (2), 192–202. 10.1093/infdis/jit450 24106295

[B18] Dowall S. D. Matthews D. A. García-Dorival I. Taylor I. Kenny J. Hertz-Fowler C. (2014). Elucidating Variations in the Nucleotide Sequence of Ebola Virus Associated with Increasing Pathogenicity. Genome Biol. 15 (11), 540. 10.1186/s13059-014-0540-x 25416632 PMC4289381

[B9] DURC Boston University (2014). Identifying and Addressing Dual Use Research of Concern. Boston: Boston University. Available from: https://www.bu.edu/researchsupport/compliance/ibc/dual-use-research-of-concern/identifying-and-addressing-dual-use-research-of-concern/ .

[B71] DURC Policy (2014a). United States Government Policy for Institutional Oversight of Life Sciences Dual Use Research of Concern. Washington, DC: United States Government.

[B72] DURC Policy (2014b). Implementation of the U.S. Government Policy for Institutional Oversight of Life Sciences DURC: Case Studies. Washington, DC: United States Government.

[B20] DURC Tools (2014). Tools for the Identification, Assessment, Management, and Responsible Communication of Dual Use Research of Concern: A Companion Guide to the United States Government Policies for Oversight of Life Sciences Dual Use Research of Concern. Bethesda, MA: National Institutes of Health.

[B81] EBRF (2016). Working Paper: Securing Immaterial Technology with Dual-Use Potential: European Biosecurity Regulators Forum (EBRF). Available from: http://www.ebrf.eu/documents/EBRF_workingpaper_210616.pdf .

[B21] EBRF (2020). Biosecurity Resource Toolbox. European Biosecurity Regulators Forum. Available from: http://ebrf.eu/toolbox.html .

[B22] Edwards D. A. Hanes J. Caponetti G. Hrkach J. Ben-Jebria A. Eskew M. L. (1997). Large Porous Particles for Pulmonary Drug Delivery. Science 276 (5320), 1868–1872. 10.1126/science.276.5320.1868 9188534

[B23] Ehrenberg R. (2015). Engineered Yeast Paves Way for home-brew Heroin. Nature 521 (7552), 267–268. 10.1038/251267a 25993934

[B24] FAS (2016). Case Studies Dual-Use. Federation of American Scientist. Available from: https://fas.org/biosecurity/education/dualuse/index.html .

[B2] Flemish Interuniversity Council (2017). Guidelines for Researchers on Dual Use and Misuse of Research. Ad hoc Working Group Dual Use of the Flemish Interuniversity Council. Available from: https://www.uhasselt.be/documents/DOC/2017VLIR003_FolderOnderzoek_EN_DEF_20180212.pdf .

[B25] Fossati E. Narcross L. Ekins A. Falgueyret J.-P. Martin V. J. J. (2015). Synthesis of Morphinan Alkaloids in *Saccharomyces cerevisiae* . PLoS One 10 (4), e0124459. 10.1371/journal.pone.0124459 25905794 PMC4408053

[B26] Gao P. Ma S. Lu D. Mitcham C. Jing Y. Wang G. (2020). Prudently Conduct the Engineering and Synthesis of the SARS-CoV-2 Virus. Synth. Syst. Biotechnol. 5 (2), 59–61. 10.1016/j.synbio.2020.03.002 32296735 PMC7156160

[B6] German Ethics Council (2014). Biosecurity - Freedom and Responsibility of Research. Berlin: German Ethics Council. Available from: https://www.ethikrat.org/fileadmin/Publikationen/Stellungnahmen/englisch/opinion-biosecurity.pdf .

[B27] GHSA (2020). Key Messages from the Global Health Security Agenda Action Package Prevent-3: Biosafety & Biosecurity. Global Health Security Agenda. Available from: Available at: https://ghsagenda.org/wp-content/uploads/2020/07/ghsa_2pager_final_print-app3.pdf .

[B29] Grange Z. L. Goldstein T. Johnson C. K. Anthony S. Gilardi K. Daszak P. (2021). Ranking the Risk of Animal-To-Human Spillover for Newly Discovered Viruses. Proc. Natl. Acad. Sci. U S A. 118 (15). 10.1073/pnas.2002324118 PMC8053939 33822740

[B30] Herfst S. Schrauwen E. J. A. Linster M. Chutinimitkul S. de Wit E. Munster V. J. (2012). Airborne Transmission of Influenza A/H5N1 Virus between Ferrets. Science 336 (6088), 1534–1541. 10.1126/science.1213362 22723413 PMC4810786

[B31] IGEM (2018). iGEM project nanoFACTORY. Bielefeld. Available from: http://2018.igem.org/Team:Bielefeld-CeBiTec .

[B19] iGEM Team Bielefeld (2015). Dual Use report by iGEM Team Bielefeld-CeBiTec. Bielefelt. Available from: http://2015.igem.org/wiki/images/b/be/Bielefeld-CeBiTec_Dual-Use_Report.pdf .

[B35] IHR (2005). Joint external evaluation tool: International Health Regulations (2005). second edition. Geneva: World Health Organization.

[B32] Imai M. Watanabe T. Hatta M. Das S. C. Ozawa M. Shinya K. (2012). Experimental Adaptation of an Influenza H5 HA Confers Respiratory Droplet Transmission to a Reassortant H5 HA/H1N1 Virus in Ferrets. Nature 486 (7403), 420–428. 10.1038/nature10831 22722205 PMC3388103

[B33] Imperiale M. J. Casadevall A. (2020). Rethinking Gain-Of-Function Experiments in the Context of the COVID-19 Pandemic. mBio 11 (4). 10.1128/mBio.01868-20 PMC7419723 32769091

[B34] Imperiale M. J. Casadevall A. (2015). A New Synthesis for Dual Use Research of Concern. Plos Med. 12 (4), e1001813. 10.1371/journal.pmed.1001813 25874461 PMC4397073

[B36] ISO (2019). ISO 35001:2019 Biorisk Management for Laboratories and Other Related Organisations. Geneva, Switzerland: International Organization for Standardization ISO.

[B13] IWG (2020). Culture of Biosafety, Biosecurity, and Responsible Conduct in the Life Sciences (Self) Assessment Framework. International Working Group on Strengthening the Culture of Biosafety, Biosecurity, and Responsible Conduct in the Life Sciences. Canada: Stimson Center and Global Affairs Canada. Available at: https://absa.org/wp-content/uploads/2020/02/Culture_of_Biosafety-Biosecurity_Self-Assessment_Framework.pdf .

[B64] IWG (2021). Establishing a Global Culture of Biosafety, Biosecurity, and Responsible Conduct in the Life Sciences: Assistance Support Initiative. Stimson Center and Global Affairs Canada. Available from: https://1540assistance.stimson.org/project/biological-threat-reduction-program-btrp/international-working-group-on-strengthening-the-culture-of-biosafety-biosecurity-and-responsible-conduct-in-the-life-sciences/ .

[B37] Jonas B. Sandbrink S. M. Joshua T. M. (2020). Widening the Framework for Regulation of Dual-Use Research in the Wake of the COVID-19 Pandemic. Washington, DC: Nuclear Threat Initiative.

[B38] Kerr P. J. Perkins H. D. Inglis B. Stagg R. McLaughlin E. Collins S. V. (2004). Expression of Rabbit IL-4 by Recombinant Myxoma Viruses Enhances Virulence and Overcomes Genetic Resistance to Myxomatosis. Virology 324 (1), 117–128. 10.1016/j.virol.2004.02.031 15183059

[B39] KNAW (2009). A Code of Conduct for Biosecurity. Amsterdam: Royal Netherlands Academy of Arts and Sciences.

[B40] KNAW (2013). Improving Biosecurity: Assessment of Dual-Use Research. Amsterdam: Royal Netherlands Academy of Arts and Sciences.

[B42] Lentzos F. Koblentz G. D. (2021). Mapping Maximum Biological Containment Labs Globally. London: King's College. Available from: https://www.globalbiolabs.org/policy-brief .

[B41] Lewis G. Millett P. Sandberg A. Snyder‐Beattie A. Gronvall G. (2019). Information Hazards in Biotechnology. Risk Anal. 39 (5), 975–981. 10.1111/risa.13235 30419157 PMC6519142

[B43] Mellata M. Ameiss K. Mo H. Curtiss R. 3rd (2010). Characterization of the Contribution to Virulence of Three Large Plasmids of Avian Pathogenic Escherichia coli χ7122 (O78:K80:H9). Infect. Immun. 78 (4), 1528–1541. 10.1128/iai.00981-09 20086082 PMC2849417

[B44] Menachery V. D. Yount B. L. Jr. Debbink K. Agnihothram S. Gralinski L. E. Plante J. A. (2015). A SARS-like Cluster of Circulating Bat Coronaviruses Shows Potential for Human Emergence. Nat. Med. 21 (12), 1508–1513. 10.1038/nm.3985 26552008 PMC4797993

[B45] Meulenbelt S. E. van Passel M. W. J. de Bruin A. van den Berg L. M. Schaap M. M. Rutjes S. A. (2019). The Vulnerability Scan, a Web Tool to Increase Institutional Biosecurity Resilience. Front. Public Health 7, 47. 10.3389/fpubh.2019.00047 30915326 PMC6422864

[B46] NASEM (2017). Dual Use Research of Concern in the Life Sciences: Current Issues and Controversies. National Academies of Sciences, Engineering, and Medicine; Policy and Global Affairs; Committee on Science, Technology, and Law; Committee on Dual Use Research of Concern: Options for Future Management. Washington (DC): National Academies Press. 29001489

[B28] NASEM (2018). Governance of Dual Use Research in the Life Sciences: Advancing Global Consensus on Research Oversight: Proceedings of a Workshop. Washington, DC: National Academies of Sciences, Engineering, and Medicine.

[B48] Nguyen Y. Jesudhasan P. R. Aguilera E. R. Pfeiffer J. K. (2019). Identification and Characterization of a Poliovirus Capsid Mutant with Enhanced Thermal Stability. J. Virol. 93 (6). 10.1128/JVI.01510-18 PMC6401428 30567995

[B47] NIH (2021). Dual Use Research of Concern. Bethesda, MA: National Institutes of Health. Available from: https://osp.od.nih.gov/biotechnology/dual-use-research-of-concern/ .

[B49] Noyce R. S. Lederman S. Evans D. H. (2018). Construction of an Infectious Horsepox Virus Vaccine from Chemically Synthesized DNA Fragments. PLoS One 13 (1), e0188453. 10.1371/journal.pone.0188453 29351298 PMC5774680

[B7] NRC (2004). Biotechnology Research in an Age of Terrorism. Washington (DC): National Research Council.

[B60] NRC (2007). Science and Security in a Post 9/11 World. A Report Based on Regional Discussions between the Science and Security Communities. Washington, DC: National Research Council. 10.17226/12013 20669431

[B50] NTI (2021). NTI Virtual Global Biosecurity Dialogue. Overcoming Challenges, Assessing Progress, and Setting Trajectories. Commitments. Washington (DC): Nuclear Threat Initiative. Available from: https://media.nti.org/documents/Virtual_GBD_Closing_Plenary_Actions_FINAL_as_of_3.5.pdf .

[B51] Oye K. A. Esvelt K. Appleton E. Catteruccia F. Church G. Kuiken T. (2014). Regulating Gene Drives. Science 345 (6197), 626–628. 10.1126/science.1254287 25035410

[B52] Peters A. (2018). The Global Proliferation of High-Containment Biological Laboratories: Understanding the Phenomenon and its Implications. Rev. Sci. Tech. OIE 37 (3), 857–883. 10.20506/37.3.2892 30964462

[B10] PHAC (2018). Canadian Biosafety Guideline - Dual-Use in Life Science Research. Ottawa: Public Health Agency of Canada. Available from: https://www.canada.ca/en/public-health/programs/consultation-biosafety-guideline-dual-use-life-science-research/document.html .

[B53] Pohanka M. Kuča K. (2010). Biological Warfare Agents. EXS 100, 559–578. 10.1007/978-3-7643-8338-1_17 20358696

[B54] Pomerantsev A. P. Staritsin N. A. Mockov YuV V. Marinin L. I. (1997). Expression of Cereolysine AB Genes in Bacillus Anthracis Vaccine Strain Ensures protection against Experimental Hemolytic Anthrax Infection. Vaccine 15 (17-18), 1846–1850. 10.1016/s0264-410x(97)00132-1 9413092

[B56] Reeves R. G. Voeneky S. Caetano-Anollés D. Beck F. Boëte C. (2018). Agricultural Research, or a New Bioweapon System? Science 362 (6410), 35–37. 10.1126/science.aat7664 30287653

[B57] RKI (2013). Hausverfügung: Dual-Use-Potenzial in der Forschung. Verfahrensregel zur Vermeidung und Minimierung von Risiken. Berlin, Germany: Robert Koch Institute. Available from: https://www.rki.de/DE/Content/Forsch/Dual-Use-Risiken/hausverfuegung.htm .

[B58] Rosengard A. M. Liu Y. Nie Z. Jimenez R. (2002). Variola Virus Immune Evasion Design: Expression of a Highly Efficient Inhibitor of Human Complement. Proc. Natl. Acad. Sci. 99 (13), 8808–8813. 10.1073/pnas.112220499 12034872 PMC124380

[B59] Sarwar S. Ilyas S. Khan B. A. Lohman D. C. Haffez S. Rafique M. (2019). Awareness and Attitudes of Research Students toward Dual-Use Research of Concern in Pakistan: A Cross-Sectional Questionnaire. Health Security 17 (3), 229–239. 10.1089/hs.2019.0002 31206321

[B61] Scudellari M. (2019). Self-destructing Mosquitoes and Sterilized Rodents: the Promise of Gene Drives. Nature 571 (7764), 160–162. 10.1038/d41586-019-02087-5 31289403

[B62] Selgelid M. J. (2009). Governance of Dual-Use Research: an Ethical Dilemma. Bull. World Health Org. 87 (9), 720–723. 10.2471/blt.08.051383 19784453 PMC2739909

[B63] Sijnesael P. C. C. van den Berg L. M. Bleijs D. A. Odinot P. de Hoog C. Jansen M. W. J. C. (2014). Novel Dutch Self-Assessment Biosecurity Toolkit to Identify Biorisk Gaps and to Enhance Biorisk Awareness. Front. Public Health 2, 197. 10.3389/fpubh.2014.00197 25368863 PMC4202726

[B65] Suffert F. Latxague É. Sache I. (2009). Plant Pathogens as Agroterrorist Weapons: Assessment of the Threat for European Agriculture and Forestry. Food Sec. 1, 221–232. 10.1007/s12571-009-0014-2

[B66] Sweere J. M. Van Belleghem J. D. Ishak H. Bach M. S. Popescu M. Sunkari V. (2019). Bacteriophage Trigger Antiviral Immunity and Prevent Clearance of Bacterial Infection. Science 363 (6434). 10.1126/science.aat9691 PMC6656896 30923196

[B67] Thi Nhu Thao T. Labroussaa F. Ebert N. V’kovski P. Stalder H. Portmann J. (2020). Rapid Reconstruction of SARS-CoV-2 Using a Synthetic Genomics Platform. Nature 582 (7813), 561–565. 10.1038/s41586-020-2294-9 32365353

[B68] Tucker J. B. (2012). Innovation, Dual Use, and Security: Managing the Risks of Emerging Biological and Chemical Technologies. The MIT Press.

[B69] Tumpey T. M. Basler C. F. Aguilar P. V. Zeng H. Solórzano A. Swayne D. E. (2005). Characterization of the Reconstructed 1918 Spanish Influenza Pandemic Virus. Science 310 (5745), 77–80. 10.1126/science.1119392 16210530

[B70] Udani R. A. Levy S. B. (2006). MarA-like Regulator of Multidrug Resistance in Yersinia pestis. Antimicrob. Agents Chemother. 50 (9), 2971–2975. 10.1128/aac.00015-06 16940090 PMC1563561

[B73] Wang W. Sun J. Wang N. Sun Z. Ma Q. Li J. (2020). Enterovirus A71 Capsid Protein VP1 Increases Blood-Brain Barrier Permeability and Virus Receptor Vimentin on the Brain Endothelial Cells. J. Neurovirol. 26 (1), 84–94. 10.1007/s13365-019-00800-8 31512144 PMC7040057

[B74] Whitby S. Novossiolova T. Walther G. Dando M. (2015). Preventing Biological Threats: What You Can Do. A Guide to Biological Security Issues and How to Address Them. University of Bradford: Bradford Disarmament Research Centre, 446p.

[B75] WHO (2006). Biorisk Management: Laboratory Biosecurity Guidance. Geneva: World Health Organization. Available at: https://apps.who.int/iris/handle/10665/69390 .

[B78] WHO (2019). WHO Benchmarks for International Health Regulations (IHR) Capacities. Geneva: World Health Organization.

[B76] WHO (2020a). Laboratory Biosafety Manual Fourth edition. Geneva: World Health Organization.

[B77] WHO (2020b). Laboratory Biosafety Manual Fourth Edition: Biosafety Programme Management. Geneva: World Health Organization.

[B82] WHO (2021). Emerging Technologies and Dual-Use Concerns: A Horizon Scan for Global Public Health. Geneva: World Health Organization. Available from: https://www.who.int/publications/i/item/9789240036161 .

[B79] Wintle B. C. Boehm C. R. Rhodes C. Molloy J. C. Millett P. Adam L. (2017). A Transatlantic Perspective on 20 Emerging Issues in Biological Engineering. Elife 6. 10.7554/eLife.30247 PMC5685469 29132504

[B80] Wollert T. Heinz D. W. Schubert W.-D. (2007). Thermodynamically Reengineering the Listerial Invasion Complex InlA/E-Cadherin. Proc. Natl. Acad. Sci. 104 (35), 13960–13965. 10.1073/pnas.0702199104 17715295 PMC1955803

[B55] Zilinskas R. A. (2017). Yersinia pestis, Biological Warfare, and Bioterrorism. New Delhi: CBW Magazine 10 (1).

